# PD-L1 Inhibitors as Monotherapy for the First-Line Treatment of Non-Small-Cell Lung Cancer in PD-L1 Positive Patients: A Safety Data Network Meta-Analysis

**DOI:** 10.3390/jcm10194583

**Published:** 2021-10-04

**Authors:** María Rosario García Campelo, Edurne Arriola, Begoña Campos Balea, Marta López-Brea, José Fuentes-Pradera, Javier de Castro Carpeno, Carlos Aguado, Diego Pérez Parente, Fidel de Oro Pulido, Pedro Ruiz-Gracia, Delvys Rodríguez-Abreu

**Affiliations:** 1 Medical Oncology, University Hospital A Coruña (XXIAC-SERGAS), 15006 A Coruña, Spain; 2 Medical Oncology, Hospital Universitari del Mar-CIBERONC, 08003 Barcelona, Spain; earriola@psmar.cat; 3 Medical Oncology, Hospital Universitario Lucus Augusti, 27003 Lugo, Spain; bcamposbalea@hotmail.com; 4 Medical Oncology, Hospital Marqués de Valdecilla, 39008 Santander, Spain; marta.lopezbrea@gmail.com; 5 Medical Oncology, Hospital Universitario Nuestra Señora de Valme, 41014 Sevilla, Spain; fuentespradera@hotmail.com; 6 Medical Oncology, Hospital Universitario La Paz, IdiPAZ, 28029 Madrid, Spain; javierdecastro5@gmail.com; 7 Medical Oncology, Hospital Clínico San Carlos, 28040 Madrid, Spain; carlos.aguado84@gmail.com; 8 Medical Affairs Department, Roche Farma S.A., 28042 Madrid, Spain; diego.perez@roche.com (D.P.P.); fidel.de_oro-pulido@roche.com (F.d.O.P.); pedro.ruiz.pr1@roche.com (P.R.-G.); 9 Medical Oncology, Hospital Universitario Insular de Gran Canaria, 35016 Las Palmas de Gran Canaria, Spain; delvysra@yahoo.com

**Keywords:** non-small cell lung cancer, network meta-analysis, immunotherapy, first-line treatment, PD-L1 inhibitors, safety

## Abstract

This network meta-analysis (NMA) evaluates the safety of first-line programmed death-ligand 1 (PD-L1) inhibitor monotherapy in advanced NSCLC patients compared to platinum-based chemotherapy. We also compared the risk of adverse events (AEs) according to programmed cell death-1 receptor (PD-1) or PD-L1 inhibitors therapy. To that end, we conducted a series of metanalyses (MAs) using data from six phase III clinical trials, including 4053 patients. Our results show a reduced risk of any grade treatment-related AEs (risk ratio (RR) = 0.722 95% CI: 0.667–0.783, *p* = 0.002), and grade 3–5 AEs (RR = 0.406 95% CI: 0.340–0.485, *p* = 0.023) in immunotherapy as compared to chemotherapy. In contrast, a higher risk of immune-related AEs (irAEs) was estimated for immunotherapy versus chemotherapy. The subgroup MAs comparing PD-L1 to PD-1 inhibitors, determined a lower risk of AEs leading to treatment discontinuation in the anti-PD-L1 subgroup (RR = 0.47 95% CI: 0.29–0.75, *p* = 0.001); however, this statistically significant difference between anti-PD-L1 and anti-PD-1 subgroups was not reached for other safety outcomes analyzed. In conclusion, our findings show that PD-L1 inhibitor monotherapy improves safety outcomes in the 1L treatment of advanced NSCLC patients as compared to chemotherapy except for irAEs.

## 1. Introduction

Lung cancer remains the leading cause of cancer death, with an estimated 1.8 million deaths worldwide accounting for 18% of total cancer deaths [1]. Non-small cell lung cancer (NSCLC) includes a variety of different lung cancers, most notably adenocarcinoma, squamous cell carcinoma, and large cell carcinoma [2]. NSCLC is the most frequent lung carcinoma, accounting for 80–90% of all diagnosed lung cancer cases [3]. With respect to NSCLC prognosis, it is dependent on the tumor, node, metastasis staging, the performance, status and concomitant comorbidities of the patient [2]. Poor 5-year survival rates have been reported for NSCLC patients in the United States between 2008 and 2014 [4].

For decades, chemotherapy has been the therapeutic strategy available for lung cancer [5]; however, in recent years, the introduction of novel agents and the use of predictive biomarkers have resulted in improved outcomes for patients with advanced/metastatic NSCLC [4]. Specifically, the use of targeted therapy with tyrosine kinase inhibitors improved patient management and their survival rates [6]. In turn, the emergence of immunotherapy, with reduced overall toxicity and non-specific side effects compared to chemotherapy and other classic cancer therapies, has been a great leap forward [7,8]. As a matter of fact, current evidence indicates that immunotherapy’s efficacy (overall survival, objective response rate and progression free survival) is superior to traditional standard chemotherapy in first line treatment for some types of cancer [8,9,10]. Moreover, treatment of advanced solid-organ malignancies with immunotherapy compared with traditional chemotherapy is associated with a lower risk of adverse events (AEs) [11]. However, immunotherapy presents specific toxicity profiles depending on its mechanisms of action [7,8,12,13].

Specifically, immunotherapy targeting programmed cell death-1 (PD-1) and programmed death-ligand 1 (PD-L1) has considerably improved the overall survival of patients, not only in those with metastatic NSCLC, but also in patients with locally advanced disease and extensive-stage small-cell lung cancer [4,14,15,16,17,18,19,20,21]. PD-L1 is expressed on tumor cells and tumor-infiltrating immune cells [4], and on activated T cells, the binding of PD-L1 to its receptor PD-1, lowers the T cell immune responses and prevents elimination of tumor cells [22,23,24,25]. Further to the central role of PD-L1 as a key element of current immunotherapy strategies, it can be used as a biomarker to predict which NSCLC patients are more likely to respond to immunotherapy [26,27,28]. A recent network meta-analysis (NMA) evaluated the efficacy of the available anti-PD-L1-containing immunotherapy strategies in monotherapy for the first-line treatment of patients with high PD-L1 expression (≥50%) and locally advanced or metastatic NSCLC. In this study, anti-PD-L1 monotherapy resulted in significantly longer overall survival and progression free survival in advanced NSCLC patients with high PD-L1 expression compared to chemotherapy alone, thus supporting the potential of this therapeutic option as a first-line strategy for this subgroup of patients [9]. In the past few years, several studies have focused on the efficacy and safety of PD-1/PD-L1 inhibitor agent immunotherapies [7,8,9,29,30,31,32,33,34,35,36,37,38]. However, no safety comparisons evaluating first-line monotherapy with anti-PD-L1 agents in NSCLC patients with a PD-L1 positive expression enriched design have been published to date. Therefore, the lack of head-to-head studies or indirect comparisons between trials, makes choosing the safest immunotherapy treatment still challenging in this patient setting.

In this study, we performed a NMA to evaluate the safety of first-line PD-L1 inhibitors monotherapy in advanced NSCLC positive PD-L1 patients compared to platinum-based chemotherapy. Moreover, we analyzed clinical trial safety outcomes comparing the anti-PD-L1 versus the anti-PD-1 treatments. Finally, we carried out indirect comparisons between immunotherapies to assess the potentially differential risk of clinically relevant immune-related AEs (irAEs).

## 2. Materials and Methods

### 2.1. Search Strategies and Study Selection

In a previous study, a systematic search was conducted in PubMed to identify all suitable trials until 1 November 2020 with no start limit applied [9]. Literature search terms used were “non-small cell lung cancer” (or “NSCLC”), “PD-L1”, “PD-1”, “pembrolizumab”, “nivolumab”, “atezolizumab”, “durvalumab”, “cemiplimab”, and all terms related to clinical trial registration (ClinicalTrials.gov, EU Clinical Trials Register, ISRCTN and ANZCTR). Additionally, a search for abstracts presented at meetings or conferences was carried out, these included: the World Conference on Lung Cancer (WCLC), the American Society of Clinical Oncology (ASCO), the American Association for Cancer Research for Medical Oncology (AACR), and the European Society for Medical Oncology (ESMO). The same literature search was applied for this safety analysis.

Only phase III randomized clinical trials (RCTs) evaluating the safety of first-line anti-PD-L1 monotherapy in patients with stage IIIB/stage IV NSCLC were included, in this way we compared homogenous populations. Studies conducted in subsets of patients already included in their corresponding pivotal trials were excluded. Observational studies, editorials, reviews, and commentaries were also ruled out. The safety data for this NMA corresponds to the as-treated populations from the six phase III RCTs that met the selection criteria. As shown in Table 1, the as-treated population included patients with different PD-L1 expression levels and all these patients, regardless their PD-L1 expression level, were analyzed in the NMA.

### 2.2. Statistical Analysis

We conducted a NMA comparing the safety estimates of all immunotherapy treatments against the common comparator, platinum-based chemotherapy. The following analyses were carried out: (1) MAs comparing the safety outcomes of all immunotherapies against chemotherapy; (2) subgroup MAs to compare safety outcomes in the PD-L1 inhibitor immunotherapies subgroup versus the PD-1 inhibitor subgroup; and (3) indirect comparisons of immunotherapies for individual clinically relevant irAEs.

Risk ratios (RR) were used as the summary estimates of relative treatment safety and were calculated along with their corresponding 95% confidence intervals (CIs) and statis-tical significance for the following safety outcomes: any grade treatment-related AEs (trAEs); grade 3–4 trAEs; grade 5 trAEs; AEs leading to discontinuation; any grade irAEs; grade 3–4 irAEs; and four specific irAEs deemed as clinically relevant by our expert physicians panel (hypothyroidism, pneumonitis, increased transaminases and nephritis). AEs were defined in the same way as in the RCTs included in this study, and their grade and severity were reported according to the National Cancer Institute Common Terminology for Adverse Events (CTCAE). Treatment safety effects are presented in forest plots by increasing risk order as compared to chemotherapy. When the 95% CI of the overall estimate does not include the unit value, the result can be considered significant at the 0.05 significance level.

For direct comparisons, MA corresponding to the analysis of binary data of proportions were performed using a DerSimonian–Laird random effects model without trans-formed proportion. The Bucher method [39] was used for adjusted indirect comparisons.

For the subgroup MAs ((anti-PD-L1)/(anti-PD-1)), the point estimate of the relative risk between subgroups was obtained by indirect comparisons. The statistical significance of the relative risk between the results of each subgroup is performed by meta-regression (omnibus *p*-value).

The results of indirect comparisons of immunotherapies for the selected irAEs are presented in league table format, which includes, for each pair of comparisons, the RR between treatments and their 95%CI. Statistical significance (*p*-value < 0.05) is established, based on the 95%CI when these do not include the unit. Summary league tables were generated for all indirect comparisons.

Heterogeneity of effect-size estimates from the individual studies was assessed with Cochran’s Q test and the I^2^ index. In this regard, a high level of heterogeneity was considered if I^2^ > 50%. Statistical significance was reached for *p*-values < 0.05, *p*-values boundaries were not controlled for multiplicity, and overall alpha was not allocated to the different analyses.

The NMA was performed using Open Meta Analyst v. 10 (Center for Evidence Syn-thesis in Health, Brown University, Providence, Rhode Island, United States). Heterogeneity between studies must be considered as guidance only due to the relatively low number of trials included in this NMA [40]. Recommendations of the Cochrane Collaboration and the Preferred Reporting Items for Systematic Reviews and Meta-Analyses (PRISMA) guidelines were followed for this MA [41].

## 3. Results

### 3.1. Studies Included in the NMA

**Figure 1:**
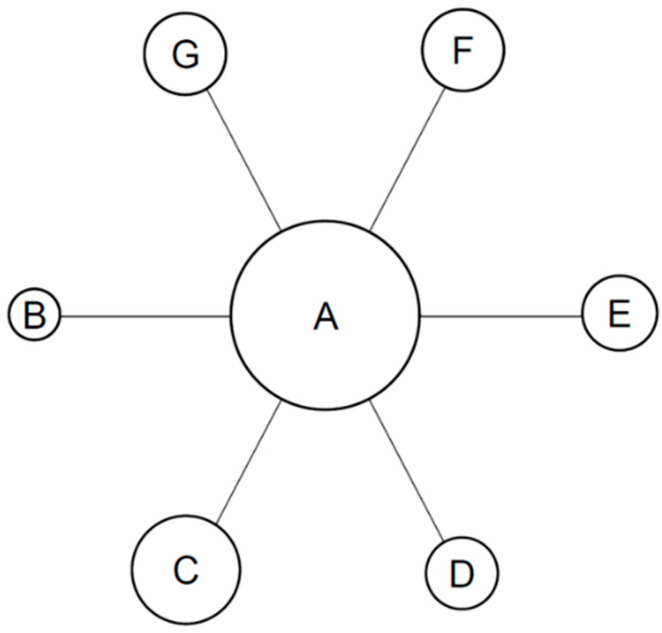
Star shaped network diagram generated in this safety MA. Only direct comparisons of immunotherapies versus overall chemotherapy as common comparator are shown. A = common comparator, platinum-based chemotherapy control group; B = KEYNOTE-024 (pembrolizumab); C = KEYNOTE-042 (pembrolizumab); D = CheckMate 026 (nivolumab); E = IMpower110 (atezolizumab); F = MYSTIC (durvalumab) and G = EMPOWER-Lung 1 (cemiplimab). Circle size is proportional to the number of patients receiving the specific treatment in each clinical trial included in the NMA.

A total of 79 records from PubMed were screened. Only six RCTs met the inclusion criteria and were analyzed. These studies included four RCTs comparing PD-1 antibody immunotherapy versus platinum-based chemotherapy: KEYNOTE-024 [20,42,43] and KEYNOTE-042 [19,44] analyzing pembrolizumab; EMPOWER-Lung 1 [45] assessing cemiplimab; and CheckMate 026 [46] analyzing nivolumab. In addition, data from two clinical trials comparing PD-L1 inhibitors versus chemotherapy were also included in our study: IMpower110 [47] and MYSTIC [48], which analyzed atezolizumab and durvalumab respectively. A total of 4053 patients monitored for AEs were included in this NMA. The flowchart for study selection is depicted in Figure S1. Comparisons of each immunotherapy treatment safety data versus the overall chemotherapy safety data generated a connected star-shaped network (Figure 1).

### 3.2. Study Characteristics

The specific characteristics of the phase III RCTs included in this NMA are summarized in Table 1. There are two methodological differences in the cemiplimab clinical trials. First, in EMPOWER-Lung 1, 31.9% of patients in the cemiplimab arm who responded to cemiplimab monotherapy could continue the drug plus treatment with four cycles of chemotherapy in the event of progressive disease under discretion of the Principal Investigator [45]. Second, studies on cemiplimab did not include a never-smoker population. It is also worth noticing that in KEYNOTE-024 [20,42,43], EMPOWER-Lung 1 [45], and CheckMate-026 [46] crossover was permitted. Patients with epidermal growth factor receptor (EGFR) or anaplastic lymphoma kinase (ALK) mutations were excluded from all the studies according to the eligibility criteria. All the studies included patients with squamous and non-squamous disease, stratified according to their histology [9]. Additionally, all studies included metastatic patients, except for KEYNOTE-042 [18,42] and EMPOWER-Lung 1 [45], which also included locally advanced NSCLC patients.

### 3.3. Safety Outputs of the NMA Comparing Immunotherapies vs. Chemotherapy

**Figure 2:**
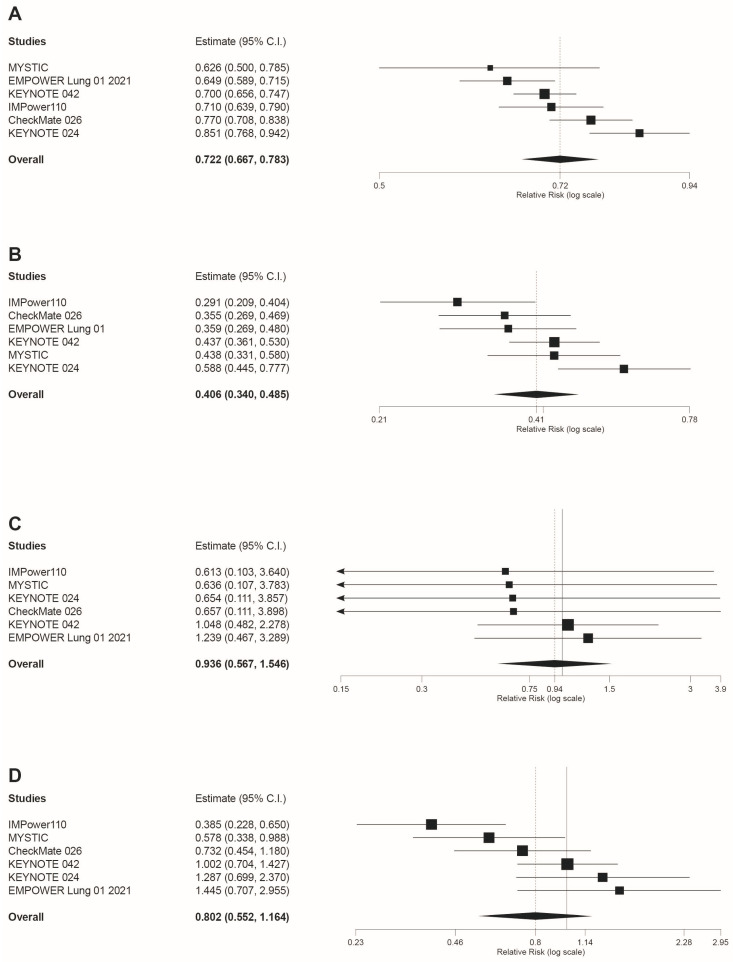
Forest plots of risk ratios (RR) for: (**A**) any grade trAEs; (**B**) trAEs grade >3; (**C**) Grade 5 trAEs; and (**D**) AEs leading to treatment discontinuation in patients treated with anti-PD-1 or anti-PD-L1 immunotherapy compared to platinum-based chemotherapy. Estimate, RR; CI, confidence interval. Treatments are ordered from top to bottom by lower to higher RR in each MA. Black squares indicate the weight of each RCT in the MA. The black rhomb indicates the weighted overall RR for immunotherapy versus chemotherapy. The I^2^ index values measuring the heterogeneity of effect-size estimates from the individual studies in MAs A to D correspond respectively to: (I^2^ = 74.15%, *p* = 0.002); (I^2^ = 61.78%, *p* = 0.023); (I^2^ = 0%, *p* = 0.954); and (I^2^ = 67.83%, *p* = 0.008).

The MA of the as-treated populations from the six phase III RCTs included in our study, revealed a statistically significant reduced risk of any grade trAEs for immuno-therapy versus chemotherapy (RR = 0.722 95%CI: 0.667, 0.783, *p* < 0.001; Figure 2A).

Likewise, immunotherapy showed a statistically significant lower risk of trAEs grade 3–4 (RR = 0.406 95%CI: 0.340, 0.485, *p* < 0.001), with atezolizumab displaying the lowest RR among the immunotherapies included in the NMA (RR = 0.291 95%CI: 0.209, 0.404; Figure 2B). However, no statistically significant differences were found between immunotherapies and chemotherapy for grade 5 trAEs (RR = 0.936 95%CI: 0.579, 1.546, *p* = 0.796; Figure 2C). As depicted in Figure 2D, when AEs leading to treatment discontinuation were analyzed, no statistically significant differences were found in the meta-analyzed data (RR = 0.802 95%CI: 0.552, 1.164, *p* = 0.2458), and only atezolizumab and durvalumab showed a significant reduced risk versus chemotherapy with atezolizumab ranking first (RR = 0.385 95%CI: 0.228, 0.650).

**Figure 3:**
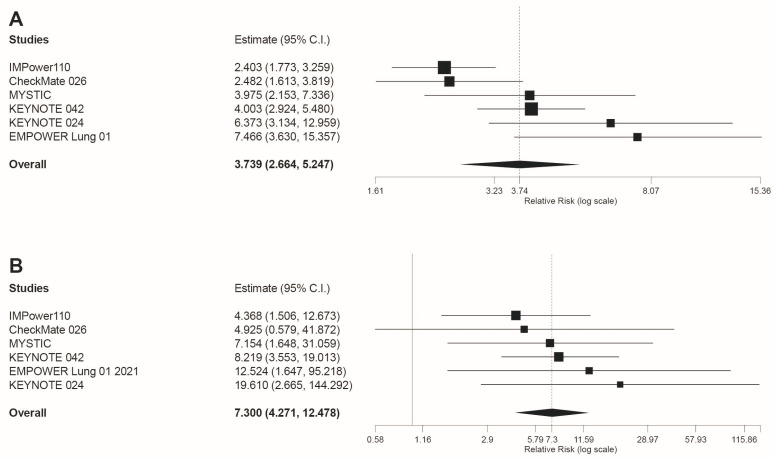
Forest plot of pooled risk ratios (RR) for (**A**) any grade irAEs and (**B**) grade 3–4 irAEs, in patients treated with anti-PD-1 or anti-PD-L1 immunotherapy compared to platinum-based chemotherapy. Estimate, RR; CI: confidence interval. Treatments are presented top to bottom by lower to higher RR. Black squares indicate the weight of each RCT in the MA. The black rhomb indicates the weighted overall RR for immunotherapy versus chemotherapy. The I^2^ index values measuring the heterogeneity of effect-size estimates from the in-dividual studies in MAs A and B correspond respectively to: (I^2^ = 68.48%, *p* = 0.007) and (I^2^ = 0%, *p* = 0.804).

Regarding irAEs, this was the only safety outcome in which a higher risk of AEs for immunotherapy compared to chemotherapy was revealed. Specifically for any grade irAEs our results were RR = 3.739 95%CI: 2.664, 5.247, *p* < 0.001 (Figure 3A). Similarly, statistically significant differences were found when irAEs grade 3–4 were analyzed (RR = 7.3 95%CI: 4.271 12.478, *p* < 0.001; Figure 3B).

The specific RR of four irAEs deemed as clinically relevant by our expert panel (hypothyroidism, pneumonitis, transaminases increased, and nephritis) were calculated. Overall, a higher risk of these selected irAEs was observed for the immunotherapies versus chemotherapy, however these differences were statistically significant only for: hypothyroidism (any grade); pneumonitis (any grade and grade 3–4); transaminases increased (grade 3–4); and any grade nephritis (Supplementary Figure S2).

### 3.4. Subgroup Analyses

**Figure 4:**
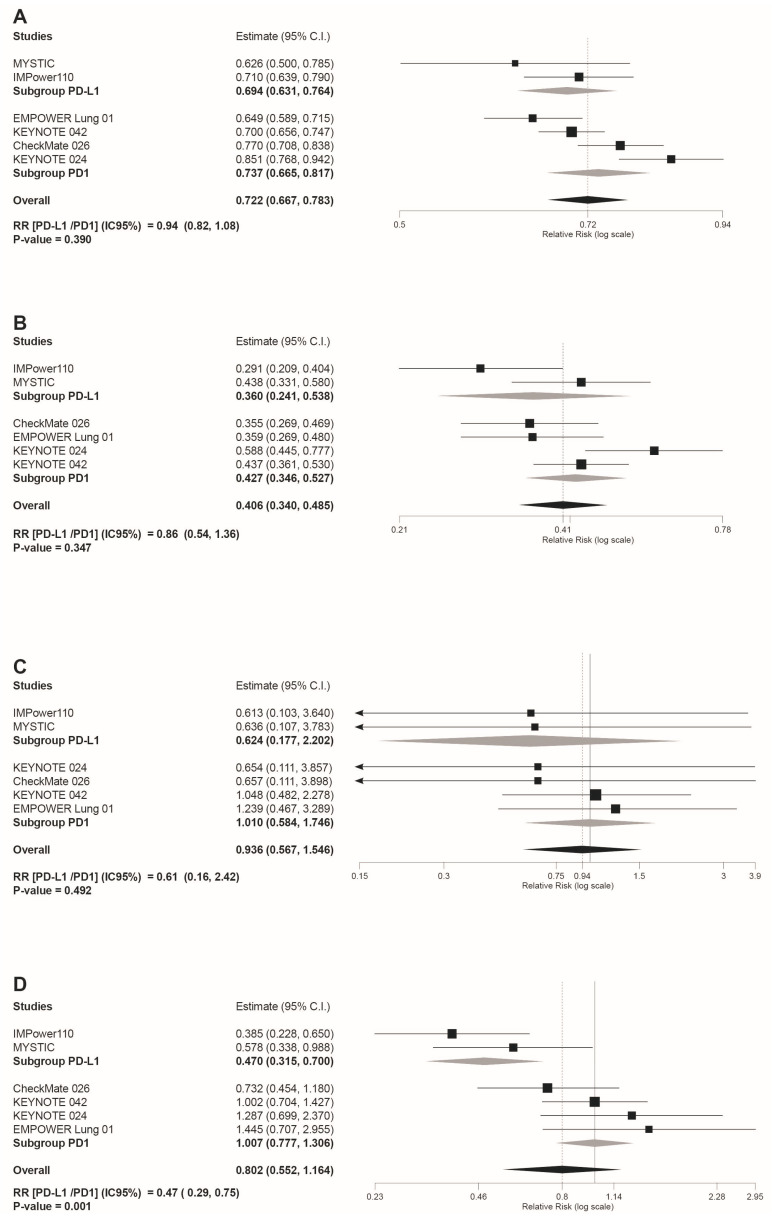
Forest plot of pooled risk ratios (RR) in the subgroup analysis((anti-PD-L1) versus (PD-1 inhibitors)) for: (**A**) any grade trAEs; (**B**) grade 3–4 trAEs (**C**) grade 5 trAEs; (**D**) AEs leading to discontinuation. Estimate, RR; CI, confidence interval. RR of ((anti-PD-L1) versus (PD1 inhibitors)) is shown at the bottom left. Treatments are ordered from top to bottom by lower to higher RR. Black squares indicate the weight of each RCT in the MA. The black rhomb indicates the weighted overall immunotherapy RR versus chemotherapy. Grey rhombs indicate, respectively, the weighted RR for anti-PD-1 immunotherapy subgroup compared to chemotherapy, and the weighted RR for anti-PD-L1 immunotherapy subgroup versus chemotherapy. The I^2^ index values measuring the heterogeneity of effect-size estimates from the individual studies in MAs A to D correspond respectively to: (I^2^ = 74.15%, *p* = 0.002); (I^2^ = 61.78%, *p* = 0.023); (I^2^ = 0%, *p* = 0.954); and (I^2^ = 67.83%, *p* = 0.008).

**Figure 5:**
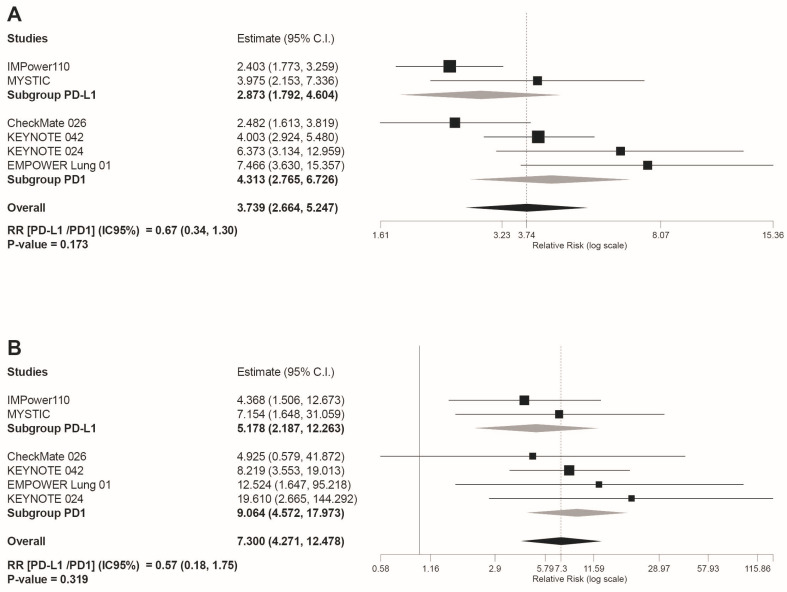
Forest plot of pooled risk ratios (RR)in the subgroup analysis((anti-PD-L1) versus (PD-1 inhibitors)) for: (**A**) any grade irAEs; and (**B**) grade 3–4 irAEs Estimate, RR; CI, confidence interval. RR of ((anti-PD-L1) versus (PD1 inhibitors)) is shown at the bottom left. Treatments are ordered from top to bottom by lower to higher RR. Black squares indicate the weight of each RCT in the MA. The black rhomb indicates the weighted overall immunotherapy RR versus chemotherapy. Grey rhombs indicate, respectively, the weighted RR for anti-PD-1 immunotherapy subgroup compared to chemotherapy, and the weighted RR for anti-PD-L1 immunotherapy subgroup versus chemotherapy. The I^2^ index values measuring the heterogeneity of effect-size estimates from the individual studies in MAs A and B correspond respectively to: (I^2^ = 68.48%, *p* = 0.007) and (I^2^ = 0%, *p* = 0.804).

Safety subgroup analyses were carried out according to the immunotherapy inhibitor target ((anti-PD-L1 subgroup) versus (anti-PD-1 subgroup)). As shown in Figure 4D, the anti-PD-L1 subgroup showed a statistically significant reduced risk for AEs leading to treatment discontinuation (RR(anti-PD-L1/PD-1 inhibitors) = 0.47 95%CI: 0.29, 0.75, *p* = 0.001). However, despite the overall reduced risk tendency of trAEs and irAEs in the PD-L1 subgroup indicated by the light grey rhombs depicted in the forest plots included in Figure 4 and Figure 5, no statistically significant differences were estimated for these safety outcomes: any grade trAEs (RR(anti-PD-L1/PD-1 inhibitors) = 0.94 95%CI: 0.82, 1,08, *p* = 0.39); grade 3–4 trAEs (RR(anti-PD-L1/PD-1 inhibitors)= 0.86 95%CI: 0.54, 1.36, *p* = 0.347); grade 5 trAEs (RR(anti-PD-L1/PD-1 inhibitors) = 0.61 95%CI: 0.16, 2.42, *p* = 0.492); any grade irAEs (RR(anti-PD-L1/PD-1 inhibitors) = 0.67 95%CI: 0.34, 1.30, *p* = 0.173); and grade 3–4 irAEs (RR(anti-PD-L1/PD-1 inhibitors) = 0.57 95%CI: 0.18, 1.75, *p* = 0.319).

In fact, for AEs leading to treatment discontinuation, we observed that the anti-PD-L1 subgroup showed a statistically significant lower risk not only versus PD-1 inhibitors, but also compared to chemotherapy (Figure 4D; RR = 0.47 95%CI: 0.315, 0.700).

Regarding individual clinically relevant irAEs, no statistically significant differences were found between the risks in the anti-PD-L1 and the anti-PD-1 subgroups (Figure S3).

### 3.5. Indirect Comparisons for Clinically Relevant irAEs

We also conducted indirect comparisons between immunotherapies to ascertain putative differences in the risk of these individual irAEs. Results are shown in league table format (Tables S1–S8). For pneumonitis (any grade), a statistically significant lower risk was observed in the durvalumab treatment versus pembrolizumab (data from the KEYNOTE-042 clinical trial; RR = 0.09 95%CI: 0.03, 0.3; Table S3). Additionally, for transaminases increased any grade, a lower risk of this AE was observed in the comparison between pembrolizumab (data from the KEYNOTE-042 RCT) and the other 4 immunotherapies (Table S5). However, these differences were not observed for pneumonitis grade 3–4, transaminases increased grade 3–4, or in the comparison of the pembrolizumab dataset from the KEYNOTE-024 RCT, and the other immunotherapy agents (Tables S3–S6).

## 4. Discussion

The data published to date suggests that the first-line immunotherapy in monotherapy strategy has become the new standard of care in locally advanced and metastatic NSCLC patients with high PD-L1 expression levels and no EGFR and ALK genomic tumor aberrations targetable mutations [9]. Currently, pembrolizumab, atezolizumab and cemiplimab have received Food and Drug Agency (FDA) and European Medicines Agency (EMA) approval as first-line monotherapy in this NSCLC patient setting. These approvals were based on the trial results from the KEYNOTE-024 [20,42,43], KEYNOTE-042 [19,44], IM-power-110 [47] and EMPOWER-Lung 1 [45] studies, respectively. Given the available therapies for NSCLC, direct or indirect comparisons between RCTs testing available immunotherapies for the treatment of NSCLC, are key to help physicians in choosing the most efficacious and safest immunotherapy treatments.

While other MAs have evaluated the efficacy and/or safety of anti-PD-L1-containing strategies [7,8,9,29,30,31,32,33,34,35,36,37,38], to date this is the first safety MA to include studies evaluating first-line single immunotherapy agents in NSCLC patients with a PD-L1 positive expression enriched design.

In the comparison of PD-L1 or PD-1 targeted immunotherapy versus chemotherapy, our MA shows a reduced risk of trAEs in immunotherapy with atezolizumab displaying the lowest risk of high grade trAEs. In line with these observations, the treatment of advanced solid-organ malignancies with immunotherapy is associated with a lower risk of AEs compared with traditional chemotherapy [11]. However, no statistically significant differences were found for grade 5 trAEs or for AEs leading to treatment discontinuation. On the other hand, in the assessment of irAEs, a higher risk was observed in immunotherapy as compared to chemotherapy. In this respect, despite the improved survival benefit associated with immune checkpoint inhibitors (ICIs), concerns of irAEs associated with ICI regimens exist because of their pharmacological mechanisms [49]. By blocking the pathways that regulate the immune system, ICIs could increase the immune system’s activity, causing organ inflammation and thus increasing the risk of irAEs [50]. Interestingly, when we analyzed four specific irAEs deemed as highly clinically relevant by our panel of expert physicians (hypothyroidism, pneumonitis, transaminases increased and nephritis), statistically significant differences between immunotherapy and chemotherapy were only found in any grade hypothyroidism, pneumonitis (any grade and grade 3–4), transaminases increased of grade 3–4, and nephritis any grade.

With respect to the subgroup MAs ([anti-PD-L1 subgroup] versus [anti-PD-1 subgroup]), Overall, a lower RR tendency for AEs in the anti-PD-L1 subgroup of IT agents was observed in the subgroup as shown by the MA forest plots. However, the RR comparison for AEs, showed no statistically significant differences between the subgroups except for AEs leading to discontinuation. In line with this result, a previously published NMA concluded that anti-PD-L1 immunotherapies may have the best safety profile in terms of both treatment-related and immune-related AEs compared to PD-1 inhibitors [13]. In fact, it is well established that anti-PD-1 and anti-PD-L1 agents exert their inhibitor activity on the PD-1/PDL1 signaling axis [23]. In this signaling pathway, PD-1 inhibitors target the PD-1 receptor, which binds to PD-L1 or PD-L2 and resists positive signals through T cell receptors, whereas anti-PD-L1 agents target the ligand PD-L1 [23,24]. Whether different outcomes in anti-PD-1 and anti-PD-L1 monotherapy could be related to their specific mechanism of action, requires further investigation. Of note, within the anti-PD-L1 agents atezolizumab demonstrated the lowest risk for AEs leading to discontinuation. Accordingly, a previous NMA and systematic review comparing different ICIs safety in NSCLC and melanoma treatment, already pointed towards a lower risk of any or high-grade AEs for atezolizumab as compared to durvalumab and the PD-1 inhibitor nivolumab [51]. In agreement, another efficacy and safety MA reported that atezolizumab was the most tolerable ICI in terms of SAEs in advance NSCLC patients as compared to pembrolizumab, nivolumab and durvalumab [7]. Regarding the subgroup MA of individual clinically relevant irAEs, no statistically significant differences were found between the risks in the anti-PD-L1 and the anti-PD-1 subgroups. Despite the lack of statistical significance, the subgroup MA forest plots obtained indicate a trend that favors PD-L1 inhibitors in the risk of pneumonitis and nephritis, whereas the opposite trend was observed regarding transaminases increased.

We also carried out indirect comparisons to evaluate differences of immunotherapy agents in the risk of individual clinically relevant irAEs. On one hand, our results indicated a slightly reduced risk of pneumonitis any grade for durvalumab compared to pembrolizumab (data from the KEYNOTE 042 clinical trial). However, this lower risk was not observed when pneumonitis grade 3–4 was studied. In line with these results, a previous systematic review reported that anti-PD1 agents showed a higher rate of irAEs and pneumonitis than PD-L1 inhibitors [31], whereas a recent MA did not find significant differences in the incidence of pneumonitis between this ICIs subgroups [30]. On the other hand, for transaminases increased any grade a lower risk was estimated for pembrolizumab (data from the KEYNOTE 042 RCT) versus the other ICIs. Again, these lower risks were not observed for transaminases increased 3–4. Intriguingly, none of these findings were observed when pembrolizumab data from the KEYNOTE-024 was compared with the rest of treatments. The fact that crossover was permitted from chemotherapy in KEYNOTE-024 [20,42,43] but not in KEYNOTE-042 [19,44] and that only patients with high PD-L1 (≥50% of TCs) expression were included in KEYNOTE-024, may have contributed to the different results observed in our MA when both pembrolizumab data sets were compared with the other immunotherapy agents.

In this study we were able to gain knowledge in the comparison of safety profiles of PD-L1 inhibitors monotherapy as 1L treatment of advanced NSCLC patients versus chemotherapy, and to underpin differences between anti-PD-1 and anti-PD-L1 immunotherapy agents, nevertheless, our NMA also has some limitations. I^2^ values > 0.5 were obtained in some of these MAs pointing out to the heterogenicity of these results. Due to the relatively low number of trials involved in this NMA, non-significant heterogeneity between studies must be considered as guidance only [40]. Additionally, our results support that single anti-PD-L1 monotherapy presents a better AE profile compared to platinum-based chemotherapy in NSCLC patients, except for irAEs, but further studies are required to assess the potential benefit/risk ratio of monotherapy versus immunotherapy combination strategies.

In conclusion, our results indicate that, except for irAEs, anti-PD-L1 monotherapy lowers the risk of AEs in the 1L treatment of advanced NSCLC patients as compared to platinum-based chemotherapy. Furthermore, the subgroup MAs showed a reduced risk of AEs leading to treatment discontinuation when compared to PD-1 inhibitors, whereas for the rest of the safety outcomes analyzed this statistical significance was not reached.

## Figures and Tables

**Table 1:** Characteristics of the studies included in the NMA.

Study	PD-L1 Expression	Experimental Arm ***	Control Arm **
KEYNOTE-024 [20,42,43]	High (≥50% of TPS)	Pembrolizumab (*n* = 154)	Platinum-based chemotherapy (*n* = 150)
EMPOWER-Lung 1 [45]	Confirmed High (≥50% of TCs) in 79,29% of patients	Cemiplimab (*n* = 355)	Platinum-based chemotherapy (*n* = 342)
IMpower110 [47]	High (≥50% of TCs or ≥10% ICs) High and intermediate (≥5% of TCs or ICs) Any expression level (≥1% of TCs or ICs)	Atezolizumab (*n* = 286)	Platinum-based chemotherapy (*n* = 283)
KEYNOTE-042 [19,44]	High (≥50% of TPS) Intermediate (≥20% of TPS) Low (≥1% of TPS)	Pembrolizumab (*n* = 636)	Platinum-based chemotherapy (*n* = 615)
MYSTIC [48]	PD-L1 ≥ 25% (assessed in TCs) ^a^ PD-L1 < 25% (assessed in TCs)	Durvalumab ± tremelimumab ^a^ (*n* = 369)	Platinum-based chemotherapy ^a^ (*n* = 352)
CheckMate 026 [46]	PD-L1 ≥ 5% (assessed in TCs) PD-L1 ≥ 1% and < 5% (assessed in TCs)	Nivolumab (*n* = 267)	Platinum-based chemotherapy (*n* = 263)

*** Number of patients in the treatment arm and ** in the control arm of the safety population (as-treated population) in each RCT. ^a^ Only the durvalumab monotherapy arm was considered for the study. PD-L1, programmed cell death-ligand 1; TCs, tumor cells; TPS, tumor proportion score. All studies enriched their populations by selecting patients according to their PD-L1 expression status: in KEYNOTE-024 [20,42,43], only patients with PD-L1 expression levels ≥50% were included; in the EMPOWER-Lung 1 trial ≥50% PD-L1 expression levels was confirmed in 563 patients [45]; regarding the IMpower-110 [47], KEYNOTE-042 [19,44], and CheckMate 026 [46] studies, patients with PD-L1 expression on at least 1% of TCs or at least 1% of tumor-infiltrating cells were included and further classified into different groups according to PD-L1 expression level. Finally, in the MYSTIC trial, patients were selected and subsequently stratified into patients with PD-L1 < 25% and PD-L1 ≥ 25%, in this RCT 25.4% of patients did not show positive PD-L1 expression [48]. Regardless of their PD-L1 expression level, all treated patients corresponding to the safety population from each trial were considered for this NMA. All RCTs included metastatic patients except for KEYNOTE-042 and EMPOWER-Lung 1, which also included locally advanced NSCLC patients.

## Data Availability

Qualified researchers may request access to individual patient level data through the clinical study data request platform (https://vivli.org/ Accessed: 30 September 2021). Further details on Roche’s criteria for eligible studies are available here (https://vivli.org/members/ourmembers/ Accessed: 30 September 2021). For further details on Roche’s Global Policy on the Sharing of Clinical Information and how to request access to related clinical study documents, see here-https://www.roche.com/research_and_development/who_we_are_how_we_work/clinical_trials/our_commitment_to_data_sharing.htm Accessed: 30 September 2021).

## Supplementary Materials

The following are available online at https://www.mdpi.com/article/10.3390/jcm10194583/s1, Figure S1: Flow chart of study selection. Figure S2: Forest plot of pooled risk ratios (RR) for clinically relevant irAEs in patients who received anti-PD-1 or anti-PD-L1 immunotherapy compared to platinum-based chemotherapy alone. Figure S3: Forest plots of pooled risk ratios (RR) for clinically relevant irAEs in the subgroup analysis (anti-PD-L1 versus PD-1 inhibitors). Table S1: League table showing the RRs and CI 95% for hypothyroidism any grade between immunotherapy treatments. Table S2: League table showing the RRs and CI 95% for hypothyroidism grade 3–4 between immunotherapy treatments. Table S3: League table showing the RRs and CI 95% for pneumonitis any grade between immunotherapy treatments. Table S4: League table including RR and CI 95% for pneumonitis grade 3–4 between immunotherapy treatments. Table S5: League table showing the RRs and CI 95% for transaminases increased any grade between immunotherapy treatments. Table S6: League table showing RR and CI 95% for transaminases increased grade 3–4 between immunotherapy treatments. Table S7: League table showing the RRs and CI 95% for nephritis any grade between immunotherapy treatments. Table S8: League table showing the RRs and CI 95% for nephritis increased any grade between immunotherapy treatments.
