# Supplementary material for: PD-L1 Inhibitors as Monotherapy for the First-Line Treatment of Non-Small-Cell Lung Cancer in PD-L1 Positive Patients: A Safety Data Network Meta-Analysis
Source: J Clin Med. 2021 Oct 4;10(19):4583. doi: 10.3390/jcm10194583 (PMC8509645; doi:10.3390/jcm10194583)
Supplement: Supplementary file 1 [file jcm-10-04583-s001.zip › jcm-1368525-supplementary.pdf]

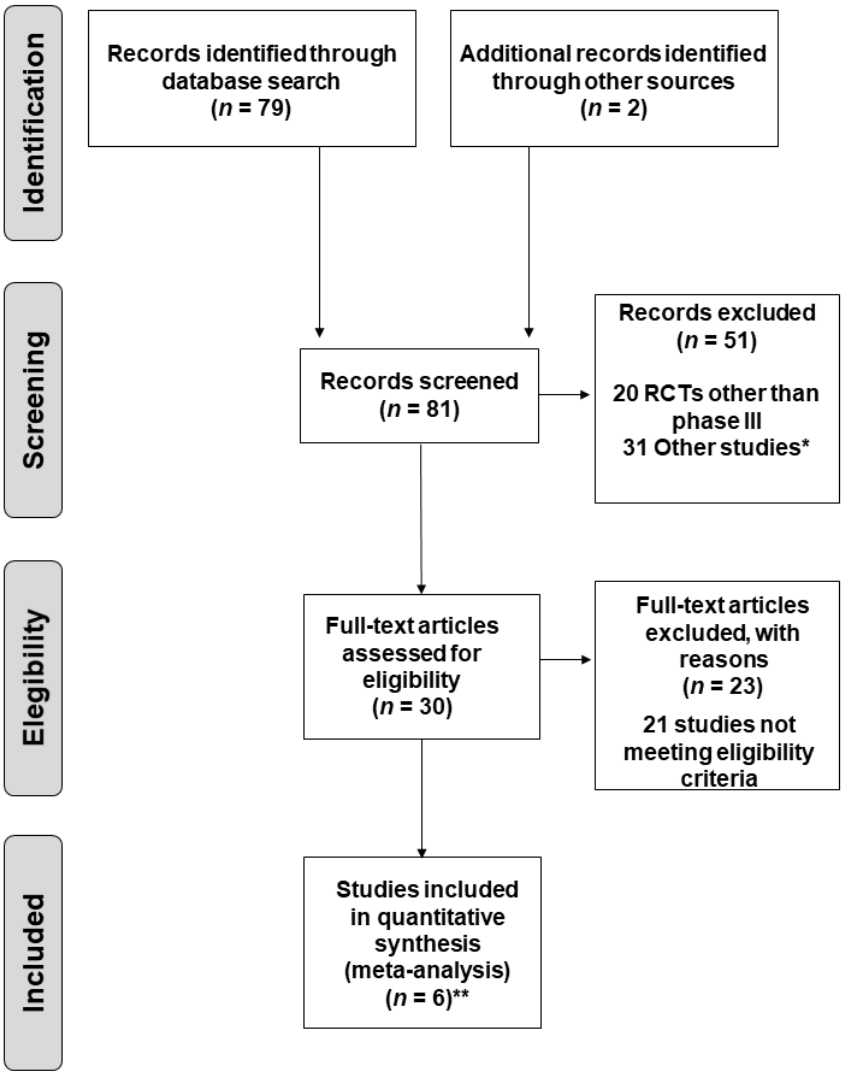

Figure S1. Flow chart of study selection [8]. RCTs = randomized controlled trials * Other studies included pooled analyses, post-marketing studies, clinical trial protocols, patient-reported outcome assessments and any study on biomarkers/gene profiling. ** Updates published for the clinical trials KEYNOTE-024 [19,39,41] and KEYNOTE-042 [18,42] were considered in the analysis.

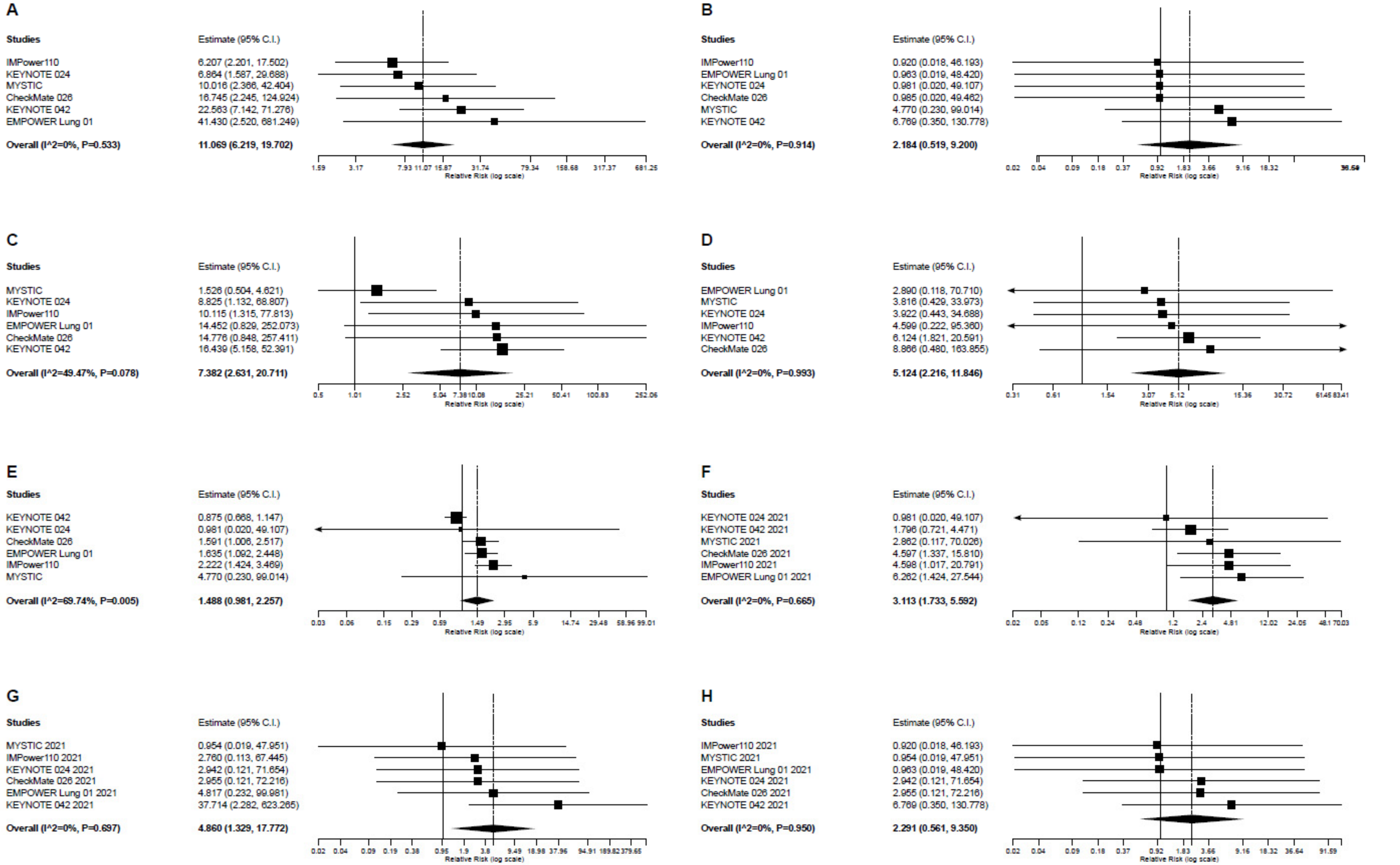

Figure S2. Forest plot of pooled risk ratios (RR) for clinically relevant irAEs in patients who received antiPD-1 or antiPD-L1 immunotherapy compared to platinum-based chemotherapy alone. (A) hypothyroidism any grade; (B) hypothyroidism grade 3-4 (C) pneumonitis any grade; (D) pneumonitis grade 3-4; (E) transaminases increased any grade; (F) transaminases increased grade 3-4; (G) nephritis any grade; and (H) nephritis grade 3-4. Estimate, RR; CI, confidence interval.

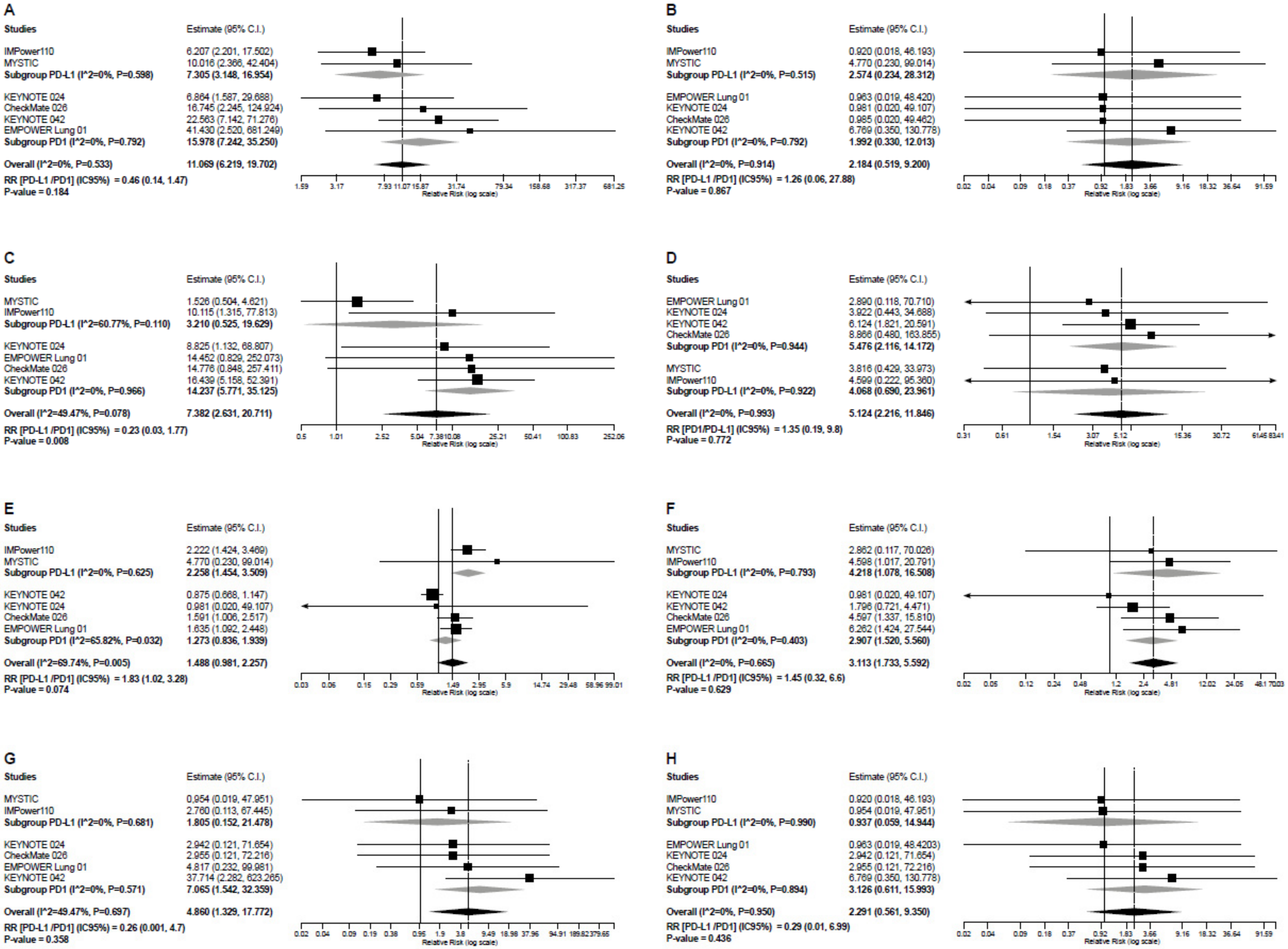

Figure S3. Forest plots of pooled risk ratios (RR) for clinically relevant irAEs in the subgroup analysis (antiPD-L1 versus PD-1 inhibitors) (A) hypothyroidism any grade; (B) hypothyroidism grade 3-4 (C) pneumonitis any grade; (D) pneumonitis grade 3-4; (E) transaminases increased any grade; (F) transaminases increased grade 3-4; (G) nephritis any grade; and (H) nephritis grade 3-4. Estimate, RR; CI, confidence interval.

Table S1. League table showing the RRs and CI 95% for hypothyroidism any grade between immunotherapy treatments.

	KEYNOTE-024	KEYNOTE-042	CheckMate-026	IMPower110	MYSTIC	EMPOWER Lung-01
**KEYNOTE-024**		3,29	2,44	0,90	1,46	6,04
Lower limit		0,51	0,20	0,11	0,19	0,26
Upper limit		21,16	29,32	7,64	11,40	142,21
**KEYNOTE-042**	0,30		0,74	0,28	0,44	1,84
Lower limit	0,05		0,07	0,06	0,04	0,09
Upper limit	1,96		7,52	1,29	4,58	37,88
**CheckMate-026**	0,41	1,35		0,37	0,60	2,47
Lower limit	0,03	0,13		0,04	0,05	0,05
Upper limit	4,93	13,65		3,56	7,10	134,10
**IMPower110**	1,11	3,64	2,70		1,61	6,67
Lower limit	0,13	0,77	0,28		0,27	0,34
Upper limit	9,34	17,10	25,88		9,54	132,14
**MYSTIC**	0,69	2,25	1,67	0,62		4,14
Lower limit	0,09	0,22	0,14	0,10		0,18
Upper limit	5,35	23,24	19,84	3,66		96,50
**EMPOWER-Lung 01**	0,17	0,54	0,40	0,15	0,24	
Lower limit	0,01	0,03	0,01	0,01	0,01	
Upper limit	3,90	11,24	21,91	2,97	5,64	

RR>1 indicates increased risk of AE for the column treatment as compared to the row treatment. Lower and upper limit of the IC95% are shown. All possible indirect comparisons are presented. Statistically significant results are highlighted in blue and underlined.

Table S2. League table showing the RRs and CI 95% for hypothyroidism grade 3-4 between immunotherapy treatments.

	KEYNOTE-024	KEYNOTE-042	CheckMate 026	IMPower110	MYSTIC	EMPOWER-Lung 01
**KEYNOTE-024**		0,70	0,14	0,14	0,15	0,14
Lower limit		0,01	0,00	0,01	0,00	0,00
Upper limit		48,85	18,70	2,84	19,44	19,45
**KEYNOTE-042**	1,42		0,19	0,21	0,21	0,20
Lower limit	0,02		0,00	0,00	0,01	0,00
Upper limit	98,38		27,69	28,58	4,34	28,79
**CheckMate 026**	7,36	5,18		1,07	1,07	1,05
Lower limit	0,05	0,04		0,00	0,00	0,02
Upper limit	1012,57	744,39		270,07	271,95	54,16
**IMPower110**	6,90	4,86	0,94		1,00	0,98
Lower limit	0,35	0,03	0,00		0,00	0,00
Upper limit	135,39	675,76	237,53		247,71	247,15
**MYSTIC**	6,87	4,84	0,93	1,00		0,98
Lower limit	0,05	0,23	0,00	0,00		0,00
Upper limit	918,19	101,82	237,24	245,70		246,86
**EMPOWER-Lung 01**	7,03	4,95	0,96	1,02	1,02	
Lower limit	0,05	0,03	0,02	0,00	0,00	
Upper limit	960,90	706,45	49,43	256,48	258,26	

RR>1 indicates increased risk of AE for the column treatment as compared to the row treatment. Lower and upper limit of the IC95% are shown. All possible indirect comparisons are presented. Statistically significant results are highlighted in blue and underlined.

Table S3. League table showing the RRs and CI 95% for pneumonitis any grade between immunotherapy treatments.

	**KEYNOTE-024**	**KEYNOTE-042**	**CheckMate 026**	**IMPower110**	**MYSTIC**	**EMPOWER-Lung 01**
**KEYNOTE-024**		1,86	1,67	1,15	0,17	1,64
Lower limit		0,18	0,05	0,06	0,02	0,05
Upper limit		19,69	56,52	20,72	1,78	55,30
**KEYNOTE-042**	0,54		0,90	0,62	0,09	0,88
Lower limit	0,05		0,04	0,06	0,03	0,04
Upper limit	**5,67**		19,64	6,43	0,30	19,21
**CheckMate 026**	0,60	1,11		0,68	0,10	0,98
Lower limit	0,02	0,05		0,02	0,00	0,02
Upper limit	20,16	24,30		22,93	2,21	40,77
**IMPower110**	0,87	1,63	1,46		0,15	1,43
Lower limit	0,05	0,16	0,04		0,01	0,04
Upper limit	15,77	16,98	48,93		1,54	47,88
**MYSTIC**	5,78	10,77	9,68	6,63		9,47
Lower limit	0,56	3,35	0,45	0,65		0,44
Upper limit	59,64	34,66	207,56	67,55		203,11
**EMPOWER-Lung 01**	0,61	1,14	1,02	0,70	0,11	
Lower limit	0,02	0,05	0,02	0,02	0,00	
Upper limit	20,62	24,86	42,61	23,45	2,26	

RR>1 indicates increased risk of AE for the column treatment as compared to the row treatment. Lower and upper limit of the IC95% are shown. All possible indirect comparisons are presented. Statistically significant results are highlighted in blue and underlined.

Table S4. League table including RR and CI 95% for pneumonitis grade 3-4 between immunotherapy treatments

	KEYNOTE-024	KEYNOTE-042	CheckMate 026	IMPower110	MYSTIC	EMPOWER-Lung 01
**KEYNOTE-024**		1,56	2,26	1,17	0,97	0,74
Lower limit		0,13	0,06	0,09	0,04	0,02
Upper limit		18,93	86,23	14,50	21,33	35,36
**KEYNOTE-042**	0,64		1,45	0,75	0,62	0,47
Lower limit	0,05		0,06	0,03	0,12	0,02
Upper limit	7,77		34,07	19,65	3,13	14,43
**CheckMate 026**	0,44	0,69		0,52	0,43	0,33
Lower limit	0,01	0,03		0,01	0,01	0,02
Upper limit	16,87	16,25		34,80	16,46	6,68
**IMPower110**	0,85	1,33	1,93		0,83	0,63
Lower limit	0,07	0,05	0,03		0,02	0,01
Upper limit	10,54	34,85	129,34		34,82	51,51
**MYSTIC**	1,03	1,60	2,32	1,21		0,76
Lower limit	0,05	0,32	0,06	0,03		0,02
Upper limit	22,53	8,06	88,88	50,57		36,44
**EMPOWER-Lung 01**	1,36	2,12	3,07	1,59	1,32	
Lower limit	0,03	0,07	0,15	0,02	0,03	
Upper limit	65,13	64,81	62,90	130,44	63,54	

RR>1 indicates increased risk of AE for the column treatment as compared to the row treatment. Lower and upper limit of the IC95% are shown. All possible indirect comparisons are presented. Statistically significant results are highlighted in blue and underlined.

Table S5. League table showing the RRs and CI 95% for transaminases increased any grade between immunotherapy treatments.

	**KEYNOTE-024**	**KEYNOTE-042**	**CheckMate 026**	**IMPower110**	**MYSTIC**	**EMPOWER-Lung 01**
**KEYNOTE-024**		0,89	1,62	2,27	4,86	1,67
Lower limit		0,02	0,03	0,04	0,03	0,03
Upper limit		44,16	81,72	115,08	675,76	83,47
**KEYNOTE-042**	1,12		1,82	2,54	5,45	1,87
Lower limit	0,02		1,07	1,51	1,46	1,15
Upper limit	55,51		3,10	4,27	20,34	3,04
**CheckMate 026**	0,62	0,55		1,40	3,00	1,03
Lower limit	0,01	0,32		0,74	0,14	0,62
Upper limit	31,07	0,94		2,65	64,36	1,71
**IMPower110**	0,44	0,39	0,72		2,15	0,74
Lower limit	0,01	0,23	0,38		0,10	0,40
Upper limit	22,43	0,66	1,36		45,99	1,34
**MYSTIC**	0,21	0,18	0,33	0,47		0,34
Lower limit	0,00	0,05	0,02	0,02		0,02
Upper limit	28,58	0,68	7,16	9,98		7,30
**EMPOWER-Lung 01**	0,60	0,54	0,97	1,36	2,92	
Lower limit	0,01	0,33	0,59	0,75	0,14	
Upper limit	30,05	0,87	1,62	2,48	62,15	

RR>1 indicates increased risk of AE for the column treatment as compared to the row treatment. Lower and upper limit of the IC95% are shown. All possible indirect comparisons are presented. Statistically significant results are highlighted in blue and underlined.

Table S6. League table showing RR and CI 95% for transaminases increased grade 3-4 between immunotherapy treatments.

	KEYNOTE-024	KEYNOTE-042	CheckMate 026	IMPower110	MYSTIC	EMPOWER-Lung 01
**KEYNOTE-024**		1,83	4,69	4,69	2,92	6,38
Lower limit		0,03	0,08	0,08	0,02	0,10
Upper limit		99,80	278,28	279,77	449,50	411,06
**KEYNOTE-042**	0,55		2,56	2,56	1,59	3,49
Lower limit	0,01		0,55	0,44	0,48	0,61
Upper limit	29,77		11,89	14,93	5,29	19,86
**CheckMate 026**	0,21	0,39		1,00	0,62	1,36
Lower limit	0,00	0,08		0,14	0,02	0,19
Upper limit	12,67	1,81		7,03	19,17	9,97
**IMPower110**	0,21	0,39	1,00		0,62	1,36
Lower limit	0,00	0,07	0,14		0,02	0,16
Upper limit	12,73	2,28	7,03		21,35	11,28
**MYSTIC**	0,34	0,63	1,61	1,61		2,19
Lower limit	0,00	0,19	0,05	0,05		0,06
Upper limit	52,81	2,08	49,46	55,11		74,18
**EMPOWER-Lung 01**	0,16	0,29	0,73	0,73	0,46	
Lower limit	0,00	0,05	0,10	0,09	0,01	
Upper limit	10,09	1,63	5,37	6,08	15,49	

RR>1 indicates increased risk of AE for the column treatment as compared to the row treatment. Lower and upper limit of the IC95% are shown. All possible indirect comparisons are presented. Statistically significant results are highlighted in blue and underlined.

Table S7. League table showing the RRs and CI 95% for nephritis any grade between immunotherapy treatments.

	KEYNOTE-024	KEYNOTE-042	CheckMate 026	IMPower110	MYSTIC	EMPOWER-Lung 01
**KEYNOTE-024**		12,82	1,00	0,94	0,32	1,64
Lower limit		0,18	0,01	0,04	0,00	0,02
Upper limit		897,45	91,87	24,83	50,68	133,71
**KEYNOTE-042**	0,08		0,08	0,07	0,03	0,13
Lower limit	0,00		0,00	0,00	0,00	0,00
Upper limit	5,46		5,50	5,14	0,43	7,95
**CheckMate 026**	1,00	12,76		0,93	0,32	1,63
Lower limit	0,01	0,18		0,01	0,00	0,05
Upper limit	91,06	896,47		85,70	50,60	51,31
**IMPower110**	1,07	13,66	1,07		0,35	1,75
Lower limit	0,04	0,19	0,01		0,00	0,02
Upper limit	28,22	959,90	98,24		54,18	143,00
**MYSTIC**	3,08	39,53	3,10	2,89		5,05
Lower limit	0,02	2,35	0,02	0,02		0,04
Upper limit	481,97	665,41	485,45	453,45		715,31
**EMPOWER-Lung 01**	0,61	7,83	0,61	0,57	0,20	
Lower limit	0,01	0,13	0,02	0,01	0,00	
Upper limit	49,88	487,47	19,31	46,95	28,06	

RR>1 indicates increased risk of AE for the column treatment as compared to the row treatment. Lower and upper limit of the IC95% are shown. All possible indirect comparisons are presented. Statistically significant results are highlighted in blue and underlined.

Table S8. League table showing the RRs and CI 95% for nephritis increased grade 3-4 between immunotherapy treatments.

	**KEYNOTE-024**	**KEYNOTE-042**	**CheckMate 026**	**IMPower110**	**MYSTIC**	**EMPOWER-Lung 01**
**KEYNOTE-024**		2,30	1,00	0,31	0,32	0,33
Lower limit		0,03	0,01	0,01	0,00	0,00
Upper limit		178,98	91,87	7,76	50,68	51,53
**KEYNOTE-042**	0,43		0,44	0,14	0,14	0,14
Lower limit	0,01		0,01	0,00	0,01	0,00
Upper limit	33,81		34,07	18,70	2,77	19,45
**CheckMate 026**	1,00	2,29		0,31	0,32	0,33
Lower limit	0,01	0,03		0,00	0,00	0,01
Upper limit	91,06	178,77		49,47	50,60	8,08
**IMPower110**	3,20	7,36	3,21		1,04	1,05
Lower limit	0,13	0,05	0,02		0,00	0,00
Upper limit	79,32	1012,57	510,38		267,00	271,31
**MYSTIC**	3,08	7,10	3,10	0,96		1,01
Lower limit	0,02	0,36	0,02	0,00		0,00
Upper limit	481,97	139,62	485,45	248,31		258,37
**EMPOWER-Lung 01**	3,06	7,03	3,07	0,96	0,99	
Lower limit	0,02	0,05	0,12	0,00	0,00	
Upper limit	480,95	960,90	76,08	247,62	253,57	

RR>1 indicates increased risk of AE for the column treatment as compared to the row treatment. Lower and upper limit of the IC95% are shown. All possible indirect comparisons are presented. Statistically significant results are highlighted in blue and underlined.
